# Self-Regeneration Performance and Characterization of Silver-Containing Activated Carbon Fibers Coated by Titanium Dioxide

**DOI:** 10.3390/polym11060983

**Published:** 2019-06-04

**Authors:** Wenjing Liu, Wang Han, Minghui Zhang, Zeyu Guo

**Affiliations:** College of Materials Science and Art Design, Inner Mongolia Agricultural University, Hohhot 010018, China; wenjing-1999@163.com (W.L.); hanwang328@163.com (W.H.)

**Keywords:** carbon materials, liquefied wood, self-regeneration, photodegradation, nanoparticles

## Abstract

In this study, wood-based activated carbon fibers (WACF) were modified by Ag nanoparticles (AgNPs) and TiO_2_ films. The coating of TiO_2_ films decreased the AgNPs agglomeration and exfoliation on WACF. As the soaking concentration of AgNO_3_ solution (*S_conc_*) increased, AgNPs size and content increased, while the pore volume (especially micropore volume) of fibers reduced. However, at higher *S_conc_* in the range of 0.2 to0.4 mol/L, only slight variations in AgNPs content and pore structure were observable for WACF/TiO_2_/Ag (Ag-containing WACF coated by TiO_2_ film). WACF/TiO_2_/Ag-0.1 (0.1 was the soaking concentration of AgNO_3_ solution, mol/L) represented the best self-regeneration performance under the visible light irradiation. The self-regeneration performance of WACF/TiO_2_/Ag was determined by the synergistic effects of two factors: adsorption and photodegradation. The abundant pores of WACF/TiO_2_/Ag-0.1 increased the methylene blue (MB) concentration of TiO_2_ surrounding and facilitated the MB photodegradation. Meanwhile, their suitable Ag content enhanced MB photodegradation. Furthermore, the principal pathway of a chemical reaction between Ag^+^ and WACF was interpreted based on the data of surface elemental constituents and surface functional groups.

## 1. Introduction

In spite of the wide use of activated carbon fibers (ACF) as the adsorption material, ACF adsorption is a costly process, and this fact has encouraged an increasing studies concern into the manufacture of low-cost alternatives to ACF as well as the regeneration process [1]. A byproduct of wood processing is sawdust, which is readily available biomass for value-added utilization. Our previous studies successfully converted sawdust into low-cost ACF in more step processes including liquefaction, melt-spinning, curing, and activation [2].

Regeneration of ACF by means of photocatalytic degradation represents great superiority because of its features of low cost and less secondary pollution in comparison with other regeneration processes such as thermal and chemical treatments, microbiological regeneration, electrochemical methods, and supercritical extraction [3]. Moreover, the photocatalytic regeneration can achieve the ACF self-regeneration by simultaneously conducting the photocatalytic degradation and adsorption [4]. 

Titanium dioxide (TiO_2_), a highly effective photocatalyst, is usually immobilized on ACF for its regeneration [4]. However, the photocatalytic degradation of TiO_2_ is partly limited because of its two inherent properties: (1) with a large bandgap (3.2 eV), TiO_2_ only absorbs UV photons, which represent only about 5% of the energy in the solar spectrum; (2) TiO_2_ has a low photocatalytic activity because of the fast recombination of charge carriers [5]. In order to extend the photoresponse region and prevent (e^−^/h^+^) pair recombination, noble metals are incorporated into TiO_2_, including Ag, Au, and Pt [6]. Compared with Au and Pt, Ag has a broader application field because of its antibacterial activity and low cost. However, in most studies, the noble metal nanoparticles are generally deposited on the surface of TiO_2_, which causes the poor stability of metals during the long-term operation. 

To overcome these drawbacks, Liu et al. prepared the TiO_2_-encapsulated Ag nanoparticles (AgNPs) supported on porous SiO_2_ bead nanospheres, which enhanced the bonding between TiO_2_ and AgNPs [5]. Moreover, it has been reported that TiO_2_ with larger surface area and smaller particle size has a stronger bonding force with ACF [7,8]. In our previous work, the weight of TiO_2_ coating on ACF varied, and the effect of TiO_2_ content on the bonding between TiO_2_ film and wood-based ACF (WACF) had been illustrated [9]. 

The aim of this research was to prepare WACF with self-regeneration performance under visible-light irradiation. The AgNPs bonding force to TiO_2_ and WACF were enhanced by coating TiO_2_ film and controlling the AgNPs size. The influence of Ag loading contents on the structure of TiO_2_/Ag-co-loaded WACF (WACF/TiO_2_/Ag) was investigated, and their self-regeneration performance was evaluated by measuring their photocatalytic degradation behavior of methylene blue (MB) under visible-light irradiation.

## 2. Materials and Methods 

### 2.1. Synthesis of Ag-Containing WACF Coated by TiO_2_ Film

To prepare the precursor fibers according to reference, 40–60 mesh wood powder from Chinese fir (*Cunninghamia lanceolata* (Lamb.) Hook.) was used [2]. The resultant precursors were heated to 800 °C with a heating rate of 5 °C/min under the nitrogen protection and activated by introducing a steam flow (1.0 mL/min) for 1 h. The obtained samples were labeled as WACF.

Furthermore, 0.5 g WACF was immersed in 60 mL AgNO_3_ (Sinopharm Chemical Reagent Co., Ltd., Beijing, China) solution over a concentration range of 0.05–0.4 mol/L at 25 °C for 24 h and then dried at 80 °C for 2 h to prepare Ag-containing WACF. 

Twenty milliliters of tetrabutyl titanate (Lynn Technology Development Co., Ltd, Shanghai, China) and 4 mL diethanolamine (Fuchen Chemical Reagent Factory, Tianjin, China) were dissolved in 24 mL ethanol (Yongsheng Chemical Reagent Factory, Tianjin, China) with stirring for 0.5 h to get solution A. Subsequently, solution B containing 12 mL ethanol, 0.4 mL acetic acid (Fuchen Chemical Reagent Factory, Tianjin, China), and 2.8 mL deionized water was slowly added into solution A with magnetic stirring at 25 °C. After the mixture was hydrolyzed for 2 h with magnetic stirring, the transparent TiO_2_ sol was obtained. 

Six grams of transparent TiO_2_ sol and Ag-containing WACF were mixed by vibration for 2 h at 25 °C. Thereafter, Ag-containing WACF coated by TiO_2_ sol was dried at 100 °C for 5 h, and then calcined at 500 °C for 1 h under nitrogen protection. The samples prepared by different AgNO_3_ solution concentrations were expressed as WACF/TiO_2_/Ag-C, where C was the soaking concentration of AgNO_3_ solution (*S_conc_*).

### 2.2. Characterization

The surface morphology of samples was observed by a field emission scanning electron microscope (FESEM, SU8010, Hitachi, Tokyo, Japan). Their elemental constituents in the selected area were detected by scanning electron microscopy coupled with energy dispersive X-ray analyzer (EDXA, 550i, IXRF, Austin, TX, USA).

The crystallite structure was analyzed by X-ray diffractometer (XRD, XRD-6000, Shimadzu, Kyoto, Japan) equipped with CuK_α_ radiation (λ = 0.154 nm). The scanning rate was 2°/min with scanning steps of 0.02° from 10° to 80° (2θ). The average crystal size (D) of Ag was calculated by the Scherrer’s formula from its (111) reflection.

The surface elemental composition and functional groups were studied by an X-ray photoelectron spectrometer (XPS, ESCALAB 250Xi, Thermo Fisher Scientific, Waltham, MA, USA). A monochromatic Al K_α_ X-ray (1486.6 eV) source served as incident radiation operated at 420 W (14 kV; 30 mA). The survey scans were collected from the binding energy (BE) of 0 to 1350 eV. XPSPEAK software was used to conduct the spectral deconvolution. A Shirley-type background was chosen and subtracted prior to quantification. After the baseline was subtracted, the curve fitting was performed with a fitting program based on an asymmetric Gaussian–Lorentzian sum function. The peak shape was optimized until an acceptable fit was obtained.

The N_2_ adsorption–desorption isotherms were measured and analyzed by a surface area and pore size analyzer (Autosorb-iQ; Quantachrome Instruments Co., Boynton Beach, FL, USA). All samples were outgassed at 300 °C for 3 h before the measurement to remove any adsorbed water or other impurities. The BET-specific surface area (*S_BET_*) was estimated via the BET equation. The total pore volume (*V_total_*) was based on the assumption that N_2_ filled the sample pores at a relative pressure (*p/p*_0_) of 0.995. The micropore area and volume (*S_micro_*, *V_micro_*) were calculated by the t-plot method. The mesopore area and volume (*S_meso_*, *V_meso_*) were estimated via the BJH method. The pore size distribution was determined by QSDFT.

### 2.3. Self-Regeneration Measurements

The self-regeneration property of samples was evaluated by adsorbing and degrading MB (CAS 7220-79-3, Jinke Fine Chemicals Co., Ltd., Tianjin, China). For this, 0.1 g samples were dispersed in 100 mL MB solution with a concentration of 200 mg/L and shaken at 25 °C. A 60-W filament lamp was used as a light source to trigger the photocatalytic reaction. After irradiation for an appropriate interval, the reaction solution was filtrated, and the concentration of MB was measured by a UV–vis spectrophotometer (TU-1950, Purkinje General Instrument Co., Ltd., Beijing, China). The change of relative absorbance was used to record the change of MB concentration in solution, that was *Ct*/C_0_ = *At*/*A_0_* (*Ct*, *At* refer to the concentration and absorbance of MB in solution at t time; *C*_0_, *A*_0_ refer to the concentration and absorbance of MB in solution at the initial time, respectively). 

## 3. Results and Discussion

### 3.1. Surface Morphology

**Figure 1:**
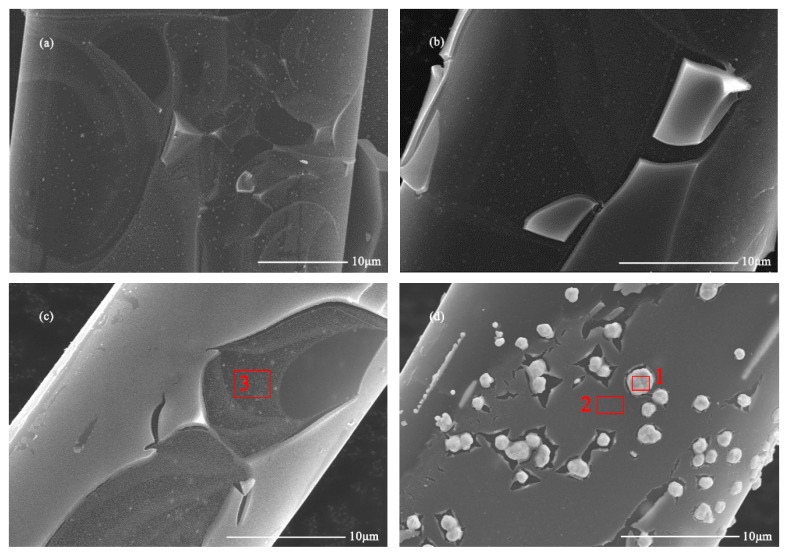
Field emission scanning electron microscope (FESEM) images of WACF/TiO_2_/Ag (Ag-containing wood-based activated carbon fibers coated by TiO_2_ film): (**a**) WACF/TiO_2_/Ag-0.05; (**b**) WACF/TiO_2_/Ag-0.1; (**c**) WACF/TiO_2_/Ag-0.2; (**d**) WACF/TiO_2_/Ag-0.4.

The FESEM images in Figure 1 show the surface morphology of WACF/TiO_2_/Ag. Numerous nanoparticles were observed on the surface of WACF, and the results of EDXA (see Table 1) proved that they were Ag nanoparticles (AgNPs). These AgNPs on the surface of WACF were coated by TiO_2_ films, and their diameters dramatically increased from about 0.2 μm to 1 μm with increasing *S_conc_*. When *S_conc_* reached 0.4 mol/L, AgNPs could not be fully covered by TiO_2_ films. Moreover, the thickness of TiO_2_ films on the fibers was not uniform. This might be attributed to the inconsistent contraction of WACF and TiO_2_ films during calcination. The contraction ratio of WACF was less than that of TiO_2_ films under the heat treatment [6]. Thus, TiO_2_ films easily split into numerous flakes during the calcination treatment [10]. Likewise, a part of TiO_2_ films with a higher thickness could fall off from fibers. Table 1 shows that element Ti was detected in areas covered by thick and thin TiO_2_ films, which demonstrates that TiO_2_ films coated all the fibers. However, the content of element Ti in the areas with thin TiO_2_ films (such as area-3) was significantly lower than that in the areas with thick TiO_2_ films (such as area-2). Element N was mainly originated from the impregnation of the AgNO_3_ solution. Thus, element N was only detectable on area 1 (on the surface of AgNPs).

### 3.2. Crystallite Structure

**Figure 2:**
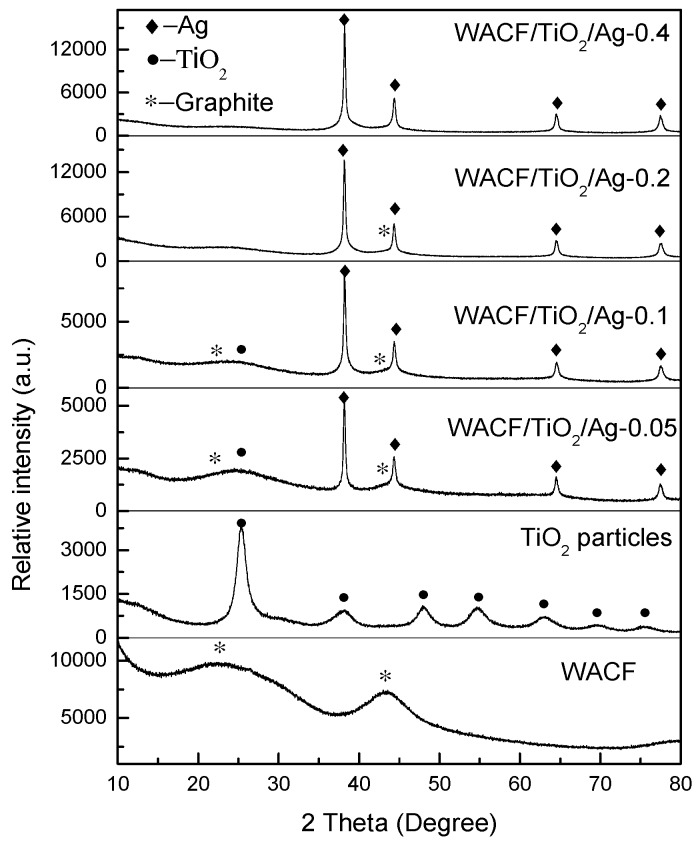
XRD patterns of WACF/TiO_2_/Ag and WACF.

XRD patterns of WACF/TiO_2_/Ag are shown in Figure 2. The four diffraction peaks of WACF/TiO_2_/Ag at 38.1°, 44.3°, 64.4°, and 77.4° are indexed to Ag, corresponding to (111), (200), (220), and (311) reflections, respectively [11]. This result indicates that most of Ag^+^ absorbed to WACF was reduced to well-crystallized metal Ag. The diffraction peak located at 25.3° is indexed to the (101) crystal plane of anatase TiO_2_ [12]. Furthermore, two broad diffraction peaks exist near 23° and 44°, which are assigned to the disordered graphitic 002 plane and 10 plane (overlapped 100 and 101), respectively [13]. However, the diffraction peaks of anatase TiO_2_ and disordered graphite became quite inconspicuous as *S_conc_* increased. This is because of the presence of amounts of metal Ag. The Ag diffraction intensity of samples prepared from higher *S_conc_* was so strong that the TiO_2_ and graphite diffraction peaks were difficult to find. Moreover, the low relative content of pure TiO_2_ led to the weak TiO_2_ diffraction peaks. TiO_2_ was prepared by the sol–gel method, and a large amount of element C existed in the gel. After the heat treatment carried out under nitrogen protection, the content of these element C in the TiO_2_ film was still large. The average crystal size (D) of Ag calculated by the Scherrer’s formula from (111) reflection is shown in Table 2. With increasing *S_conc_*, the average crystal size of Ag was slightly enlarged.

### 3.3. Surface Chemical Structure

The elemental composition of WACF/TiO_2_/Ag was analyzed by XPS, and Table 3 shows that the WACF/TiO_2_/Ag all contain C, O, Ti, Ag, and N elements. Element C was the most abundant constituent of all the samples. Compared with WACF, less C and more O were found in WACF/TiO_2_/Ag. This is because the TiO_2_ film contained large amounts of O. Certainly, the addition of new elements (such as Ti and Ag) also caused a decrease in C content. Moreover, the Ag content increased with increasing *S_conc_*, indicating that more Ag was fixed on fibers at higher *S_conc_*. 

**Figure 3:**
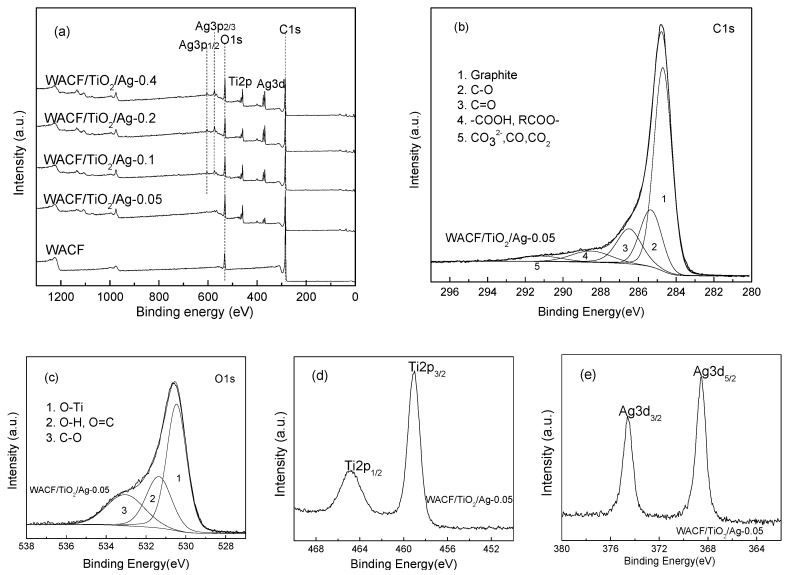
(**a**) X-ray photoelectron spectrometer (XPS) spectra of WACF/TiO_2_/Ag and WACF; (**b**–**e**) high resolution XPS spectra of the C1s, O1s, Ti2p, and Ag3d regions taken on WACF/TiO_2_/Ag-0.05.

**Figure 4:**
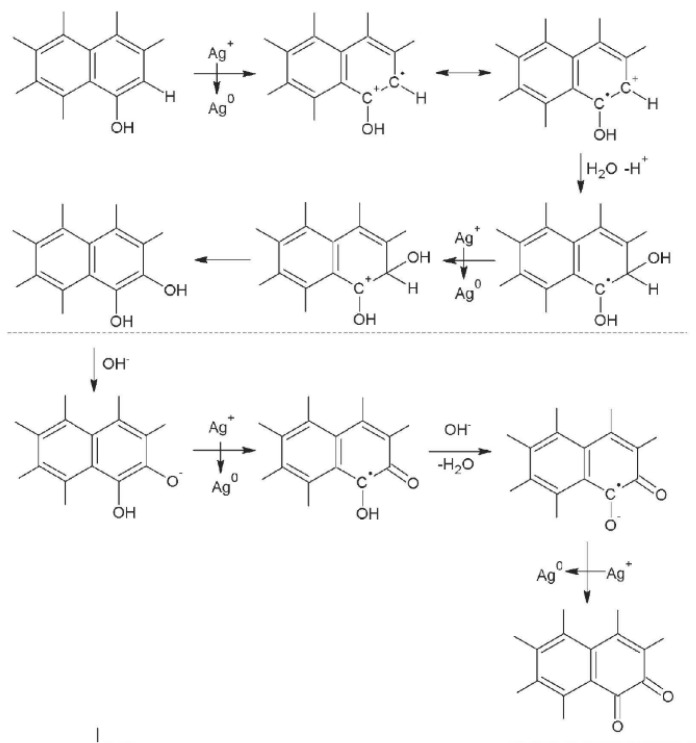
The principal pathway of chemical reaction between Ag^+^ and WACF.

To obtain information about the surface functional groups of fibers and confirm the valence states of various atoms, measurements of XPS spectra (see Figure 3a) of the C1s, O1s, Ti2p, and Ag3d regions were analyzed. The XPS spectra of these regions were similar, so only WACF/TiO_2_/Ag-0.05 is shown in Figure 3b–e as an example. As in Figure 3b, the C1s spectra exhibit an asymmetric tailing and can be deconvoluted into five peaks. These are (see Table 2): graphitic carbon (C–C, *BE* = 284.7 eV); carbon species in phenolic, alcohol, and/or ether groups (C–O, BE = 285.4 eV); carbon in carbonyl groups and/or quinine groups (C=O, *BE* = 286.5 eV); carbon in carboxyl and/or ester groups (–COOH, RCOO–, *BE* = 288.7 eV); and carbon in carbonate groups (*BE* = 291.2 eV) [2]. The graphitic carbon was the predominant component for all the samples. After loading AgNPs and TiO_2_ films, the content of graphitic carbon decreased, while the contents of C–O and C=O largely increased. This is because a part of graphitic carbon was oxidized during the process of loading AgNPs and TiO_2_ films. When the fibers were immersed in AgNO_3_ solution, with the transition from Ag^+^ to Ag^0^, the water attacked intermediate radical cation formed at locations to produce C–O where ortho or para dihydroxy groups were present [14]. As the reaction continued, C–O on catechol and hydroquinone were oxidized to generate C=O (see Figure 4). With increasing *S_conc_*, more Ag^+^ was reduced into Ag^0^, thus more graphitic carbon was oxidized into C–O. Additionally, the hydroxyl groups on the surface of TiO_2_, originating from the reaction between adsorbed H_2_O and TiO_2_, such as H_2_O + Ti–O–Ti→2Ti–OH [15], could trap the photogenerated holes (h^+^) to produce hydroxyl radicals with very strong oxidizing properties. Some graphitic carbon was involved in this oxidization reaction, also leading to the increment of C–O content.

As shown in Figure 3c and Table 3, the spectra of O1s region of WACF/TiO_2_/Ag were deconvoluted into three peaks: O–Ti bond in the TiO_2_ lattice (*BE* = 529.8–530.1 eV); O–H and O=C groups (*BE* = 530.9–531.5 eV); and O–C groups (*BE* = 532.2–532.9 eV) [16]. Another three kinds of oxygen functional groups were found on WACF with the peak at 530.9 eV corresponding to the O=C group, the peak at 532.2–532.9 eV corresponding to the O–C group, and the peak at 535.6 eV corresponding to adsorbed O_2_ or H_2_O. The disappearance of adsorbed O_2_ or H_2_O peak on the surface of WACF/TiO_2_/Ag was ascribed to the reaction between adsorbed H_2_O and TiO_2_ and the blocking of pores. The O–Ti became the main component after loading AgNPs and TiO_2_ films, while the content of O–C decreased from 71.22% to (21.57–26.09)%. Our previous research reported that the pure TiO_2_ film only contained small content of O–C (8.4%) [9]. Judging from these results and EDXA analysis, it can be inferred that TiO_2_ film in some areas of fibers was thin (<XPS detecting depth, 3 nm). Moreover, the content of O–C increased with increasing *S_conc_*, which was in good agreement with the previous result about C1s spectra.

Figure 3d shows the XPS spectrum of Ti2p doublet peaks, the binding energies of Ti2p_1/2_ and Ti2p_3/2_ are located at approximately 464.7 eV and 459.0 eV, respectively. The split between the Ti2p_1/2_ and Ti2p_3/2_ core levels was 5.7 eV, indicating a normal state of Ti^4+^ in the anatase TiO_2_ [17]. This result otherwise proves that Ag atoms did not replace Ti^4+^ to form lattice defects. It is because Ag atom size (1.26 Å) was larger than Ti atom size (0.67 Å), and it was difficult to fit into the TiO_2_ lattice [18].

In Figure 3e, the two peaks observed at 373.9 eV and 368.1 eV are according with Ag3d_3/2_ and Ag 3d_5/2_, indicating that the AgNPs loaded on the fibers mainly existed in the form of zero-valence [19]. 

### 3.4. Pore Structure

**Figure 5:**
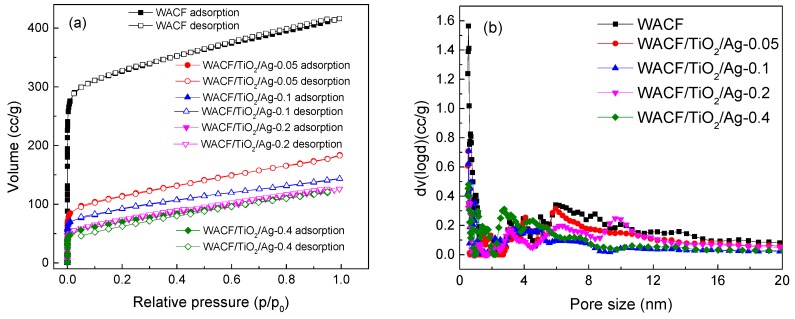
(**a**) N_2_ adsorption–desorption isotherms of WACF/TiO_2_/Ag and WACF; (**b**) Pore size distribution of WACF/TiO_2_/Ag and WACF.

Pore structure characterization is important to understand the adsorption property of WACF/TiO_2_/Ag. Figure 5a shows the N_2_ adsorption–desorption isotherms of WACF and WACF/TiO_2_/Ag. The N_2_ adsorption increased sharply at a low relative pressure (*p*/*p*_0_ < 0.1) and then gradually rose with increasing relative pressure at *p*/*p*_0_ > 0.1. The results express the coexistence of micropores and mesopores in the samples [20]. Compared with WACF, the N_2_ adsorption of WACF/TiO_2_/Ag drastically reduced. The detailed pore structure parameters are presented in Table 4. Ag and TiO_2_ loading blocked numerous pores of WACF, leading to a decrease in specific surface area (S) and pore volume (V). As *S_conc_* increased from 0.05 mol/L to 0.2 mol/L, S and V reduced sharply. However, only a slight decrease in S and V was observable when *S_conc_* continued to rise. This is because the AgNP size became larger at higher *S_conc_*, which led to a decrease in adhesion force between AgNPs and fibers. Therefore, some larger AgNPs fell off from the fibers, and the Ag content showed a slight rise in the course of *S_conc_* increment from 0.2 to 0.4 mol/L. This interpretation was also verified by FESEM images and elemental composition analysis. The comparison of micropores and mesopores shows that the decrease ratio of micropores was obviously higher than that of mesopores with increasing *S_conc_*. This result indicates that AgNPs was preferred to fill the micropores. The quoted researches demonstrate that the adsorbability of micropores to AgNPs was larger than that of mesopores [19]. Therefore, the AgNPs in mesopores could escape from the pores to the surface of the fiber and combine with other AgNPs on the surface to form large AgNPs.

The pore size distribution of WACF/TiO_2_/Ag is shown in Figure 5b. The loading of AgNPs and TiO_2_ film led to a decrease in micropore and mesopore volume. The decrease in micropore volume mainly occurred in the range of 0.5–1 nm. As *S_conc_* increased, the size of blocked mesopores was increased owing to the increasing AgNPs.

### 3.5. Self-Regeneration Performance

**Figure 6:**
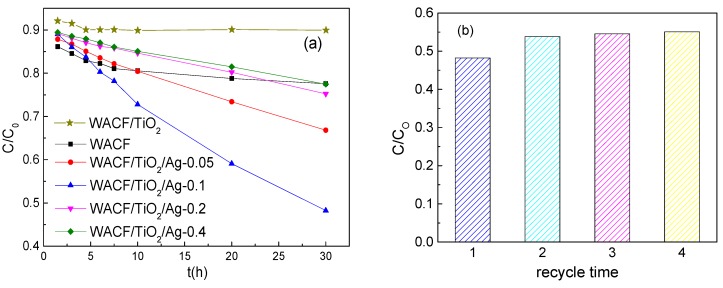
(**a**) Methylene blue (MB) removal effects of WACF/TiO_2_/Ag, WACF/TiO_2_, and WACF; (**b**) MB removal effects of WACF/TiO_2_/Ag-0.1 undergoing cyclic trials.

**Figure 7:**
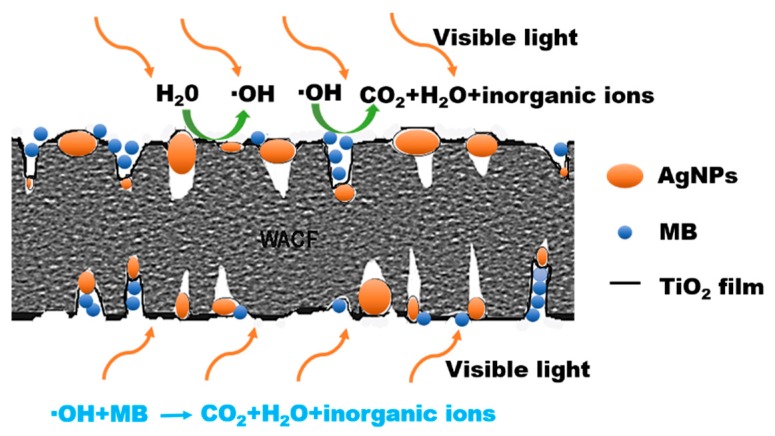
Schematic illustrations of self-regeneration mechanism of WACF/TiO_2_/Ag.

Figure 6a illustrates the MB removal effects of WACF, WACF/TiO_2_, and WACF/TiO_2_/Ag under the visible light irradiation. In the initial stage of contact (0–5 h), about 20% MB was removed by WACF due to their abundant pores. As time prolonged, the pores of WACF were blocked by MB, and their adsorption capacity nearly reached saturation. In comparison, WACF/TiO_2_/Ag kept the removal effect on MB during the whole process of the experiment, which demonstrates that WACF/Ag/TiO_2_ had the self-regeneration performance under the visible light irradiation. As Ag content increased, the self-regeneration performance of WACF/TiO_2_/Ag increased initially and then decreased. The samples prepared by 0.1 mol/L AgNO_3_ solution represented the best self-regeneration performance. The self-regeneration performance of WACF/TiO_2_/Ag was determined by the synergistic effects of two factors: adsorption and photodegradation [21]. The developed pore structure of WACF/TiO_2_/Ag could increase the MB concentration of TiO_2_ surrounding and facilitate MB photodegradation. Meanwhile, the MB photodegradation would release the blocked pores and then realize the WACF self-regeneration. The probable self-regeneration mechanism of WACF/TiO_2_/Ag is described in Figure 7. It is well known that pure TiO_2_ can be photoexcited only under the UV irradiation. The AgNPs loading enlarged the photoresponse region and enhanced the photocatalytic activity of TiO_2_. AgNPs exhibited a surface plasmonic resonance effect, which produced a strong and broad absorption in the visible light region. In addition, more photogenerated electrons were generated after AgNPs loading, and the recombination of the photogenerated electrons and holes was inhibited, both of which enhanced the photocatalytic activity of TiO_2_ [22]. The AgNPs content influenced the photocatalytic activity of TiO_2_. When the AgNPs content was insufficient, there were not enough reaction sites for enhancing the photocatalytic activity of TiO_2_. However, the excess AgNPs might act as a recombination center and decrease the efficiency of charge separation. The results of pore structure, surface morphology, and surface chemical composition analysis indicated that WACF/TiO_2_/Ag-0.1 had developed pore structure and suitable AgNPs content compared with other samples. Thus, WACF/TiO_2_/Ag-0.1 showed the best self-regeneration performance. It is remarkable that only about 10% MB was removed by WACF/TiO_2_, even lower than that removed by WACF. This is because WACF/TiO_2_ had fewer pores compared with WACF [9], and little photocatalytic degradation occurred for WACF/TiO_2_ with visible light irradiation.

In order to determine the self-regeneration durability of samples, WACF/TiO_2_/Ag-0.1 was used repeatedly for MB removal. Figure 6b shows only a slight decrease in MB removal (<10%) after the fourth trial. It means that both AgNPs and TiO_2_ films attached to fibers firmly. 

## 4. Conclusions

WACF with self-regeneration performance was successfully prepared by loading AgNPs and TiO_2_ films on their surface. AgNPs were homogeneously immobilized on WACF and coated by TiO_2_ films. This structure effectively avoided AgNPs agglomeration and exfoliation. With increasing *S_conc_*, AgNPs size and content increased, while the pore volume of fibers reduced. However, when *S_conc_* rose from 0.2 mol/L to 0.4 mol/L, only slight variations in AgNPs content and pore structure were observable. The samples prepared by 0.1 mol/L AgNO_3_ solution showed the best self-regeneration performance. The abundant pores of WACF/TiO_2_/Ag-0.1 increased the MB concentration of TiO_2_ surrounding and facilitated MB photodegradation. Meanwhile, their suitable Ag content enhanced the MB photodegradation. Moreover, the results of cyclic trials demonstrated that WACF/TiO_2_/Ag-0.1 had excellent self-regeneration durability.

## Figures and Tables

**Table 1:** The content of elemental constituents (at %) in the selected areas (see Figure 1) detected by energy dispersive X-ray analyzer (EDXA).

Selected Area	C	O	Ti	Ag	N
1	26.17	10.39	2.92	56.15	4.36
2	73.92	21.35	3.91	0.82	0.00
3	91.16	5.77	1.08	1.99	0.00

**Table 2:** The average crystal size of Ag in WACF/TiO_2_/Ag.

Sample	2θ (°)	β (°)	D (nm)
WACF/TiO_2_/Ag-0.05	38.18	0.35	23.81
WACF/TiO_2_/Ag-0.1	38.20	0.35	23.82
WACF/TiO_2_/Ag-0.2	38.16	0.32	26.04
WACF/TiO_2_/Ag-0.4	38.20	0.30	27.78

**Table 3:** Surface elemental composition (at %) and results in the fitting of C1s and O1s regions.

Sample	Content of the Element (at %)					C1s (%)					O1s (%)			
	C1s	O1s	Ti2p	Ag3d	N1s	Graphite	C–O	C=O	–COOH, RCOO–	CO_3_^2-^, CO, CO_2_	O–Ti	O–H, O=C	O–C	O_2_, H_2_O
WACF	90.79	8.89	0	0	0.32	64.11	14.89	9.88	5.91	5.21	0	20.05	71.22	8.72
WACF/TiO_2_/Ag-0.05	70.96	18.61	6.38	0.70	3.36	56.83	18.49	14.55	6.64	3.49	53.34	25.09	21.57	0
WACF/TiO_2_/Ag-0.1	73.21	16.23	5.91	1.61	3.04	56.31	19.21	12.88	6.72	4.87	52.61	25.26	22.13	0
WACF/TiO_2_/Ag-0.2	73.11	16.01	5.20	2.26	3.42	51.82	24.02	14.04	5.58	4.52	51.97	24.69	23.33	0
WACF/TiO_2_/Ag-0.4	69.46	18.18	6.52	2.30	3.54	49.92	24.68	14.13	5.37	5.89	46.36	27.55	26.09	0

**Table 4:** Specific surface area (m^2^/g) and pore volume (cm^3^/g) of WACF and WACF/TiO_2_/Ag.

Sample	Total Pores		Micropores		Mesopores	
	*S_BET_*	*V_total_*	*S_micro_*	*V_micro_*	*S_meso_*	*V_meso_*
WACF	1250	0.644	968	0.384	186	0.207
WACF/TiO_2_/Ag-0.05	411	0.285	213	0.091	143	0.162
WACF/TiO_2_/Ag-0.1	342	0.202	205	0.087	98	0.091
WACF/TiO_2_/Ag-0.2	254	0.195	105	0.048	111	0.122
WACF/TiO_2_/Ag-0.4	245	0.185	96	0.044	104	0.119
